# *BRCA*1/2 testing: therapeutic implications for breast cancer management

**DOI:** 10.1038/s41416-018-0127-5

**Published:** 2018-06-05

**Authors:** Nadine M. Tung, Judy E. Garber

**Affiliations:** 1 Beth Israel Deaconess Medical Center, 330 Brookline Avenue, Boston, MA 02215 USA; 2 Harvard Medical School, 25 Shattuck Street, Boston, MA 02115 USA; 3 Dana-Farber Cancer Institute, 450 Brookline Avenue Boston, Boston, MA 02215 USA

**Keywords:** Breast cancer, Predictive markers

## Abstract

Testing for germline *BRCA*1/2 mutations has an established predictive role in breast cancer risk assessment. More recently, studies have also identified *BRCA*1/2 status as clinically relevant in the selection of therapy for patients already diagnosed with breast cancer. Emerging breast and ovarian cancer research indicate that *BRCA* status predicts responsiveness to platinum-based chemotherapy, as well as to inhibitors of poly(ADP-ribose) polymerase (PARP), owing to the ability of these interventions to inhibit DNA repair pathways. *BRCA*1/2 mutation testing thus has important and expanding roles in treatment planning for subsets of patients with breast cancer. Recent studies have demonstrated different activity of platinum salts in *BRCA*-mutated compared with non-*BRCA*-mutated breast cancer. Furthermore, phase II/III studies of single-agent PARP inhibitors (PARPi) have shown encouraging progression-free survival results in patients with *BRCA*1/2-mutated breast cancer, which led to the recent approval of olaparib, the first PARPi to be approved in breast cancer. Determining *BRCA*1/2 mutation status in this breast cancer subgroup could potentially expand treatment options beyond the current standard of taxane and anthracycline-based chemotherapy. Although attempts have been made to develop scoring systems that measure defects in homologous recombination repair pathways to predict response to platinum or PARPi, none have yet made it into clinical use. In this review, we summarise the recent and ongoing preclinical and clinical studies on the treatment of *BRCA*-associated breast cancer, and discuss efforts to identify other breast cancer patients who may be responsive to therapies effective in *BRCA* mutation carriers, including platinum-containing chemotherapy and PARPi.

## Introduction

Germline mutations in *BRCA*1 or *BRCA*2 (g*BRCA*1/2m) confer a well-established increased risk for the development of papillary serous ovarian cancer (OC), triple-negative breast cancer (TNBC; *BRCA*1), oestrogen receptor (ER)-positive breast cancer (BC), and human epidermal growth factor receptor 2 (HER2)-negative BC (*BRCA*2); however, all BC subtypes can occur in association with g*BRCA*1/2m.^1–8^ Systemic therapies are routinely selected by a few well-established markers of response, including tumour ER and progesterone receptor (PgR) expression, and tumour amplification or overexpression of HER2.^9^ Until recently, BC cases that are negative for all three of these predictive biomarkers have been treated as a homogeneous group using chemotherapy, due to a lack of therapeutic targets and treatments specific for TNBC subtypes. Advances in molecular profiling are making it increasingly apparent that TNBCs are heterogeneous, and that certain biomarkers may predict clinical response to specific therapies.^10–13^ At this time, among the most clinically relevant of these predictive biomarkers are loss-of-function mutations in *BRCA*1 or *BRCA*2 (*BRCA*1/2), which are present in up to 20% of patients with TNBC,^14^ and these represent an opportunity for improved precision treatment. There is also growing evidence that *BRCA*1/2 mutations (*BRCA*1/2m) are clinically relevant for identifying subtypes of hormone receptor-positive HER2-negative BC.^15,16^

This review evaluates current research on the treatment of *BRCA*-associated BC, and highlights the importance of *BRCA*1/2m testing to help identify patients with BC who might benefit from platinum-based chemotherapy, as well as treatment with poly(ADP-ribose) polymerase inhibitors (PARPi). The biological and preclinical evidence supporting the rationale for targeting *BRCA*1/2m in BC are described, and evidence from recent and ongoing clinical studies with platinum salts and PARPi is highlighted in both advanced and early-stage BC settings. Finally, we briefly explore the biomarkers currently being evaluated for potential future clinical application, including a look beyond *BRCA*1/2m and the concept of *BRCA*ness.

## Who (and what) to test for *BRCA* mutation

The value of g*BRCA*1*/*2m testing for cancer risk reduction in breast and ovarian cancer is well-established.^17–19^ To date, researchers from the Breast Cancer Information Core (https://research.nhgri.nih.gov/bic/) have collated more than 1800 mutations in the *BRCA*1 gene and 2000 mutations in the *BRCA*2 gene.^20^ This collection of mutations comprises intronic changes, missense mutations, small in-frame insertions and deletions, and large rearrangements,^20^ and different mutations have been reported to confer varying risks for developing OC or BC. In a study of more than 30,000 *BRCA*1/2m carriers, researchers identified risk clusters that comprised variants of strong and potential clinical significance, as well as other variants of unknown clinical significance and benign or likely benign variants.^7^ This analysis indicated that OC cluster regions, which are associated with greater OC risk, tended to be located in the central region of each gene; whereas BC cluster mutations were located in the 5′ and 3′ regions.^7,20^ Alterations that appear to confer higher risk include missense mutations in the RING finger, *BRCA*1 C-terminal (BRCT), and DNA-binding domains, large genomic rearrangements, and founder mutations in both *BRCA* genes;^7,20^ however, validation is required before these data can be used for clinical risk assessment.

Beyond testing for g*BRCA*1/2m, a small percentage of *BRCA*-related cancers contain purely somatic mutations, which can be detected through direct analysis of the tumour tissue or circulating cell-free DNA.^21,22^ The percentage of somatic *BRCA*1/2m in BC is not well established; however, two studies found that ~3% of unselected primary BCs had a somatic mutation in *BRCA*1 or *BRCA*2.^23,24^ By contrast, 19% of 235 unselected OCs were found to harbour a somatic *BRCA*1/2m.^25^ Among the 28 ovarian tumours with a somatic *BRCA*1/2m for which a blood sample (germline DNA) was available, 11 (39%) were found not to have a germline *BRCA*1/2m, suggesting that 7%–8% of OCs harbour only a somatic *BRCA*1/2m, compared with 3% in BC.^25^ Whereas there is accumulating evidence to support testing for somatic *BRCA*1/2m to guide therapy, the clinical implications for treatment are not yet clear for BC or OC.^26^ In BC, the rationale for somatic *BRCA*1/2m testing is less developed than for germline testing, but may soon become reasonable in some settings if specific *BRCA*-targeted therapies, such as PARPi, are found to be effective in the presence of *BRCA*1/2m alone. Of note, although these mutations are uncommon in prostate cancer, data suggest that patients with somatic *BRCA*1/2m will respond to PARPi.^27^ The efficacy of PARPi for advanced BC with somatic *BRCA*1/2m is currently being investigated in a study by the Translational Breast Cancer Research Consortium (NCT03344965).

The role of *BRCA* testing to guide therapy selection in patients already diagnosed with BC is evolving. Box 1 lists the patient characteristics that should trigger testing for *BRCA*1/2m in those already diagnosed with BC. The presence of *BRCA*1/2m may offer patients additional or alternative treatment options as discussed in this review, in both early-stage and advanced BC.

Box 1. Characteristics that should trigger testing for germline *BRCA*1/2 mutation in patients already diagnosed with breast cancer

- Family history of breast, ovarian/tubal/peritoneal cancer, pancreatic, or aggressive prostate cancer
- Young age at diagnosis (<50 years)
- Triple-negative breast cancer (ER-negative, PgR-negative, and HER2-negative)
- Breast cancer in a male
- Ashkenazi Jewish heritage
- Personal history of ovarian or pancreatic cancer
- Detection of somatic *BRCA*1/2 mutation
- Patient with metastatic HER2-negative breast cancer who is eligible for treatment with a PARPi^19^

*ER-negative* oestrogen receptor-negative, *HER2-negative* human epidermal growth factor receptor 2-negative, *PgR-negative* progesterone receptor-negative

## Evidence and rationale for targeting *BRCA*1/2 mutations

The biologic rationale for targeting *BRCA*1/2 mutations with PARPi or platinum salts in BC has been supported by preclinical studies investigating these interventions in BC, combined with clinical data assessing carboplatin in OC. Wild-type *BRCA*1 and *BRCA*2 are tumour-suppressor genes and their protein products have multiple functions, including serving as key enzymes in the homologous recombination pathway, which is the high-fidelity mechanism for repair of DNA double-strand (DS) breaks.^28,29^ Loss-of-function mutations or silencing of *BRCA*1/2 disables the homologous recombination pathway, which forces cells to rely on lower fidelity more error-prone pathways for DNA repair, such as non-homologous end-joining, during which mutations and genetic instability may be introduced.^30–32^

Inhibition of single-strand (SS) DNA break repair in tumour cells with homologous recombination deficiency^33^ is synthetically lethal, and can cause cell death. Poly(ADP-ribose) polymerase-1 (PARP-1) is an integral enzyme in the repair pathway for SS DNA breaks; PARP-1 detects SS breaks and induces the creation of PAR chains on itself and adjacent nuclear proteins, thus signalling for other DNA repair enzymes to accumulate.^33^ Eventually, PARP-1 ‘PARylates’ itself, allowing it to be released from the DNA and continue its cycle of sensing SS breaks. Therefore, in *BRCA*-deficient tumours with defective homologous recombination, PARPi prevent the repair of SS DNA breaks, which may lead to the accumulation of DS breaks and stalled DNA replication forks, leading to cell death.^34^ Another way that PARPi may contribute to cell death is through the trapping of PARP proteins on DNA; the trapped PARP-1/DNA complexes can interfere with DNA replication, which also occurs by stalling the DNA replication forks.^32,35^ Preclinical data have demonstrated differences in PARP trapping potency among PARPi (talazoparib exhibited the highest potency, followed by niraparib, olaparib, and rucaparib, with veliparib having the lowest potency). Trapping potencies did not correlate with their respective PARP catalytic inhibition,^35,36^ and it is not clear whether these differences have any significant impact on clinical efficacy or safety.

The wild-type *BRCA* allele commonly undergoes loss-of-heterozygosity during tumourigenesis in individuals with heterozygous *BRCA*1/2 m, leading to severe deficiency in the homologous recombination pathway.^20^ Therefore, a germline heterozygous *BRCA*1/2 mutation may be sufficient to identify tumour cells that are more likely to be sensitive to PARPi. In support of this, PARPi strongly reduced the proliferation and survival of cells harbouring defects in homologous recombination due to deficiencies in *BRCA*1/2 in laboratory and xenograft models.^37,38^ Furthermore, high sensitivity to PARPi was observed in cells with deficiency in homologous recombination repair, including loss-of-*BRCA*1/2,^37^ or loss of another protein essential for repair of DS breaks.^39^ This is not surprising, given that a single-functional *BRCA* allele is sufficient to maintain homologous recombination.

Notably, preclinical studies investigating platinum salts found that cells lacking functional *BRCA*1 or *BRCA*2 were highly sensitive to cisplatin.^40^ This is consistent with platinum-based chemotherapy studies in OC, which reported higher response rates in patients with homologous recombination deficiencies.^41,42^ This sensitivity has been attributed to the formation of covalent crosslinks in the DNA, and an impaired ability of cells to repair this damage in the absence of competent homologous recombination.^20,43^ Improved response rates to platinum-based chemotherapy in patients with *BRCA*1/2m OC, as well as the high sensitivity of homologous recombination-deficient cells to PARPi and platinum salts, led to clinical studies investigating both of these methods for the identification of responders and prediction of patient outcomes with targeted agents in the treatment of *BRCA*-related BC, OC, and other tumours. It also stimulated the search for further biomarkers of response.

## *BRCA* mutations in advanced and metastatic BC management

Evidence regarding the use of *BRCA* status or homologous recombination competency to guide treatment selection in BC is strongest in the setting of metastatic disease, and several recent clinical studies have evaluated platinum salts and PARPi in patients with advanced or metastatic BC and *BRCA*1/2m.

### Clinical outcomes in locally advanced and metastatic BC: platinum salts

The largest study testing the ability of g*BRCA*1/2m status to predict response to platinum salts was the ‘TNT’ Triple-Negative Breast Cancer Trial.^44,45^ Enroled patients (*n* = 376) had recurrent, locally advanced or metastatic BC that was confirmed to be triple-negative or occurred in a known *BRCA*1/2m carrier (regardless of ER, PgR, or HER2 status). Patients were randomised to carboplatin or docetaxel as first-line treatment for recurrent disease, and stratified according to *BRCA*1/2 status. There was no significant difference in objective response rate (ORR), median progression-free survival (PFS), or overall survival (OS) in the overall population; however, among carriers of a *BRCA*1/2m (*n* = 43), the carboplatin group had a significantly higher ORR than the docetaxel group (68% versus 33%; *P* = 0.03), and the interaction between treatment effect and *BRCA* status was significant (*P* = 0.01).^45^ Likewise, among mutation carriers, PFS was significantly longer in the carboplatin group versus the docetaxel group (6.8 versus 4.8 months; *P* = 0.03), and the interaction between treatment effect and *BRCA* status was significant (*P* = 0.03).^46^ High-response rates to platinum salts in women with *BRCA*-related metastatic BC have also been reported in other studies, as described in Table 1.^47,48^

**Table 1:** Selected studies with platinum salts in g*BRCA*1/2m breast cancer

Study	Disease		Phase	Total (*n*)	*BRCA*m (*n*)	TNBC (*n*)	*BRCA*m ER^+^ (*n*)		Treatment	Response in overall population	Response in patients with wt*BRCA*1/2		Response in patients with g*BRCA*1/2m	Response in patients with *BRCA*1m
*Advanced and metastatic breast cancer*														
TNT^45^	Metastatic TNBC or *BRCA*1/2 mutation BC		III	376	43	363	12		Carboplatin vs docetaxel	ORR: 31% with carboplatin vs 36% with docetaxel	NR		68% with carboplatin vs 33% with docetaxel	NR
TBCRC009^48^	Metastatic TNBC		II	86	11	86	0		Cisplatin or carboplatin	ORR: 25.6% (22/86)	ORR: 19.7% (13/66)		ORR: 54.5% (6/11)	NR
NCT01611727^47^	Metastatic BC with a *BRCA*1 mutation		II	20	20^a^	14	5		Cisplatin	NR	NR		NR	ORR: 80% CR: 45%
Brocade 2 NCT01506609^82^	Locally recurrent or metastatic BC with a g*BRCA*1/2 mutation		II	99	99	42	56 (ER+ and/or PgR+)		Paclitaxel/carboplatin/placebo (PCP)	61%	NR		61%	NR
*Early-stage disease*														
NCT01630226^85^		Stage I–III BC with a *BRCA*1 mutation	II	107	107^b^	82		16	Cisplatin	NR		NR	NR	pCR: 61%
GeparSixto/GBG 66 NCT01426880^87,88^		Stage I–III TNBC or HER2+ BC	II/III	588	50	315 291 *BRCA* known		0	Backbone regimen ± carboplatin	With vs without carboplatin: TNBC pCR: 53% vs 37% 291 *BRCA* known status: 57% vs 41%		With vs without carboplatin: TNBC pCR: 55% vs 36%	With vs without carboplatin: TNBC pCR: 65% vs 67%	NR

*BC* breast cancer, *CR* complete response, *ER+* oestrogen receptor-positive, *gBRCA1/2* germline BRCA1/2, *HER2+* human epidermal growth factor receptor 2-positive, *NR* not reported, *ORR* overall response rate, *pCR* pathologic complete response, *PgR+* progesterone receptor-positive, *TNBC* triple-negative breast cancer, *wtBRCA1/2* wild-type BRCA1/2

^a^All were HER2-negative, 15 were ER-negative, 17 were PgR-negative, and 14 were TNBC

^b^100 were HER2-negative (5 HER2 status unknown), 86 were ER-negative (5 ER status unknown), 91 were PgR-negative (6 PgR status unknown), and 82 were TNBC

These studies indicate that patients with advanced or metastatic BC associated with g*BRCA*1/2m have high-clinical response rates to cisplatin and carboplatin, and those response rates are associated with longer PFS. It is not yet clear whether those clinical benefits will translate into longer OS.

### Clinical outcomes in locally advanced and metastatic BC: PARP inhibitors

PARPi have received FDA approval for a variety of indications over the last 4 years. Olaparib has recently (January 2018) received FDA approval for the treatment of patients with g*BRCA*1/2m HER2-negative metastatic BC who were previously treated with chemotherapy in neoadjuvant, adjuvant, or metastatic settings.^49^ This makes olaparib the first PARPi approved for the treatment of BC. Previously, 2014 saw the approval of olaparib for use as monotherapy in patients with deleterious or suspected deleterious g*BRCA*1/2m advanced OC who were treated with three or more prior lines of chemotherapy,^50^ and in August 2017, olaparib received approval for the maintenance treatment of adult patients with recurrent epithelial ovarian, fallopian tube, or primary peritoneal cancer who experience a complete or partial response to platinum-based chemotherapy.^51^ Furthermore, niraparib gained FDA approval in March 2017 for the maintenance treatment of adult patients with recurrent epithelial ovarian, fallopian tube, or primary peritoneal cancer who experience a complete or partial response to platinum-based chemotherapy.^52,53^ Rucaparib was approved in 2016 for treatment of patients with deleterious *BRCA* mutation-associated advanced OC who were treated with two or more chemotherapies.^54^

Olarapib was the first PARPi to undergo extensive clinical testing in BC. Antitumour responses, measured by ORR, radiologic response, or tumour-marker response, were reported in early studies of women with previously treated and advanced BC or OC.^55–57^ Later, an open-label, single-arm phase II basket study reported results for olaparib monotherapy in 62 women with *BRCA*1/2m-related advanced BC.^58^ These patients were heavily pretreated, with a mean of 4.6 chemotherapy regimens in the metastatic setting, including 42 (68%) who had received prior platinum therapy (cisplatin or carboplatin). Partial responses were observed in 8 patients (13%) and stable disease ≥8 weeks was observed in 29 patients (47%). Response rates were lower in patients who had received prior platinum therapy (9.5%; 95% confidence interval (CI): 2.7–22.6) compared with those who had not (20%; 95% CI: 5.7–43.7). Although this difference was not statistically significant, it is consistent with the concept that platinum chemotherapy and PARPi may be targeting the same mechanistic pathways (i.e., DNA repair), and therefore may have some degree of cross-resistance.^58^ A similar trend related to prior platinum chemotherapy was also observed for pancreatic and prostate cancer.^58^ Of note, response to PARPi in OC is strongly associated with responsiveness to platinum chemotherapy; one study demonstrated a 61.5% ORR (by RECIST) to single-agent olaparib in platinum-sensitive OC, but no RECIST responses in platinum-refractory OC.^59^ This concept is also supported by a phase II study in which patients with g*BRCA*1/2m-associated metastatic BC were treated with veliparib alone followed by combination with carboplatin at progression; only 1 of 30 patients responded to combination therapy after progression on veliparib.^60^ A phase II study with olaparib is summarised in Table 2.^57^

**Table 2:** Selected phase II/III studies with PARP inhibitors in g*BRCA*1/2m locally advanced and metastatic breast cancer

Study	Disease	Phase	*N*	Treatment	Efficacy in patients with g*BRCA*1/2m
NCT00494234^57^	Recurrent, advanced BC with median 3 prior regimens and *BRCA*1/2 mutation (*BRCA*1/2m)	II	27	Olaparib 400 mg BID	ORR: 41% (11/27)
OlympiAD NCT02000622^61^	Metastatic BC with g*BRCA*1/2m	III	302	Olaparib 300 mg BID vs treatment of physician’s choice (TPC; capecitabine, eribulin, or vinorelbine)	ORR: 60% with olaparib vs 29% with TPC PFS: 7.0 months with olaparib vs 4.2 months with TPC (hazard ratio 0.58; 95% CI: 0.43–0.80; *P* < 0.001) DoR: 6.4 months with olaparib (IQR, 2.8–9.7) vs 7.1 months with TPC (IQR, 3.2–12.2)
ABRAZO NCT02034916^65^	Advanced BC with g*BRCA*1/2m following platinum or multiple cytotoxic regimens	II	84	Talazoparib 1 mg/day following platinum-based therapy (cohort 1) vs ≥3 platinum-free cytotoxic-based regimens (cohort 2)	ORR: 21% (95% CI: 10–35) in cohort 1 vs 37% (95% CI: 21–55) in cohort 2 PFS: 4.0 months (95% CI: 2.8–5.4) in cohort 1 vs 5.6 months (95% CI: 5.5–7.8) in cohort 2 DoR: 5.8 months (95% CI: 2.8–NR) in cohort 1 vs 3.8 months (95% CI: 2.8–10.1) in cohort 2 CBR: 38% (95% CI: 24–53) in cohort 1 vs 66% (95% CI: 48–81) in cohort 2
EMBRACA NCT01945775^64^	Advanced BC with g*BRCA*1/2m	III	431	Talazoparib 1 mg/day vs physician’s choice of chemotherapy (PCT; capecitabine, eribulin, gemcitabine, or vinorelbine)	ORR: 63% (95% CI: 56–69) with talazoparib vs 27% (95% CI: 19–36) with PCT PFS: 8.6 months (95% CI: 7.2–9.3) with talazoparib vs 5.6 (95% CI: 4.2–6.7) with PCT DoR: 5.4 months (95% CI: 2.8–11.2) with talazoparib vs 3.1 (95% CI: 2.4–6.7) with PCT CBR24: 69% (95% CI: 63–74%) with talazoparib vs 36% (95% CI: 28–45)
BRAVO NCT01905592^66^	Metastatic BC with g*BRCA*1/2m (and HER2-negative)	III	306 (est)	Niraparib vs physician’s choice of chemotherapy	ONGOING
Cancer Research UK^67^	Previously treated advanced OC or BC with g*BRCA*1/2m	II	78 *n* = 23 (BC)	Rucaparib	39% of BC patients (9/23) achieved stable disease ≥12 weeks
RUBY NCT02505048^68^	HER2-negative metastatic BC associated with BRCAness phenotype determined by “high-tumour genomic LOH” score and/or a somatic *BRCA*m	II	41 (est)	Rucaparib	ONGOING
Brocade 2 NCT01506609^82^	Locally recurrent or metastatic BC with g*BRCA*1/2m	II	284	Paclitaxel/carboplatin/veliparib (PCV) vs paclitaxel/carboplatin/placebo (PCP)	PFS: 14.1 months with PCV vs 12.3 months with PCP; hazard ratio 0.789 (95% CI: 0.536–1.162); *P* = 0.227 ORR: 78% with PCV vs 61% with PCP; *P* = 0.027
Brocade 3 NCT02163694^83^	Locally advanced or metastatic g*BRCA*1/2m BC (and HER2-negative)	III	500 (est)	Paclitaxel/carboplatin/veliparib vs paclitaxel/carboplatin/placebo	ONGOING

*BC* breast cancer, *BID* twice daily, *CBR* clinical benefit rate, *CBR24* CBR at 24 weeks, *CI* confidence interval, *DoR* duration of response, *est* estimated, *IQR* interquartile range, *LOH* loss-of-heterozygosity, *gBRCA1/2m* germline *BRCA*1/2 mutation, *OC* ovarian cancer, *ORR* objective response rate, *PARP* poly(ADP-ribose) polymerase, *PCP* paclitaxel/carboplatin/placebo, *PCV* paclitaxel/carboplatin/veliparib, *PCT* physician’s choice chemotherapy, *PFS* progression-free survival, *TNBC* triple-negative breast cancer, *TPC* treatment of physician’s choice

More recently, the phase III OlympiAD study (NCT02000622) assessed olaparib monotherapy versus physician’s choice of chemotherapy (capecitabine, vinorelbine, or eribulin) for the treatment of metastatic BC in 302 patients with g*BRCA*1/2m (Table 2). At the December 2016 data cutoff, PFS by blinded independent central review was significantly longer with olaparib than with physician’s choice chemotherapy (7.0 versus 4.2 months; hazard ratio (HR) 0.58; 95% CI: 0.43–0.80; *P* < 0.001). An exploratory subgroup analysis of PFS was conducted, and although the study was not powered to detect differences among these subgroups, the HR favoured olaparib versus physician’s choice for patients with TNBC (HR 0.43; 95% CI: 0.29–0.63) and *BRCA*1/2m carriers of either subtype: *BRCA*1m (HR 0.54; 95% CI: 0.37–0.79) and *BRCA*2m (HR 0.68; 95% CI: 0.45–1.07).^61^ Another study also reported a doubling of ORR with olaparib versus physician’s choice chemotherapy (59.9% versus 28.8%, respectively) and greater change from baseline in target lesion size with olaparib (–45.1% versus –14.8%, respectively).^62^ A prespecified secondary analysis that examined health-related quality of life found that patients who received olaparib experienced significantly less and significantly delayed deterioration in global health-related quality of life, compared with physician’s choice chemotherapy.^63^ In a similarly designed phase III study (EMBRACA, NCT01945775), talazoparib was compared with physician’s choice of therapy (capecitabine, eribulin, gemcitabine, or vinorelbine) in 431 patients with g*BRCA*1/2m advanced BC (Table 2). Talazoparib significantly prolonged PFS by blinded independent central review (8.6 months, 95% CI: 7.2–9.3 versus 5.6 months, 95% CI: 4.2–6.7; HR 0.542; *P* < 0.0001), and delayed time to deterioration in global health status/quality of life (HR 0.38; 95% CI: 0.26–0.55; *P* < 0.0001), compared with physician’s choice of therapy.^64^ Other PARPi that are currently being evaluated in phase II/III clinical studies in advanced cancers related to *BRCA*1/2m, including BC (rucaparib, veliparib, and niraparib) are described in Table 2.^64–68^

**Fig. 1:**
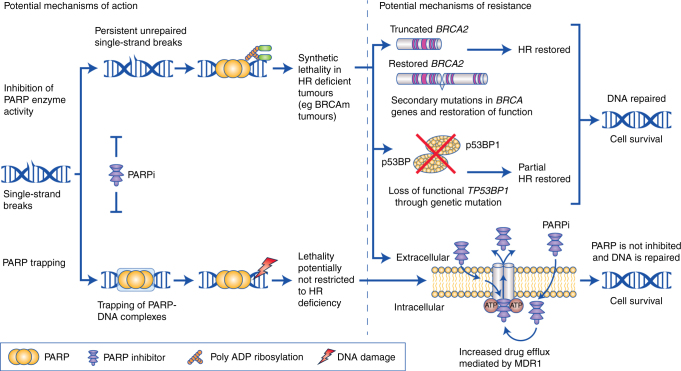
PARP inhibitors: Some possible mechanisms of action and resistance. The left panel illustrates two possible mechanisms of action of PARPi. Upper pathway: Inhibition of PARP enzyme activity or catalytic inhibition interferes with the repair of single-strand breaks, leading to stalled DNA replication forks that requires HR repair. In HR-deficient tumours, such as those with *BRCA*m, PARP inhibition results in synthetic lethality. Lower pathway: PARP trapping refers to trapping of PARP proteins on DNA, which also leads to replication fork damage, but because this pathway utilises additional repair mechanisms, it is not restricted to tumours with HR deficiency. The right panel illustrates three possible mechanisms of resistance to PARPi. These include: (1) secondary mutations in *BRCA* genes that restore *BRCA* function and HR; (2) somatic mutation of TP53BP1, causing partial restoration of HR; and (3) increased PARPi efflux mediated by MDR1/P-glycoprotein 1, preventing the drugs from acting at the appropriate sites. The first two mechanisms of resistance restore HR and apply to PARP catalytic inhibition in HR-deficient tumours; whereas, the third mechanism applies to both mechanisms of action of PARPi. *BRCA*m *BRCA* mutation; HR homologous recombination; MDR1 multidrug resistance protein 1; p53BP1 tumour suppressor p53-binding protein 1; PARP poly(ADP-ribose) polymerase; PARPi PARP inhibitor

The results from studies of PARPi in advanced and metastatic BC suggest that these agents are active against BC in women with germline *BRCA1/2* loss-of-function mutations, but response rates remain around 60%.^61^ Lack of response in *BRCA*1/2m carriers may be due to several mechanisms of resistance^69,70^ (Fig. 1). Secondary somatic mutations that restore function of *BRCA*1 or *BRCA*2 have been reported to predict clinical resistance to PARPi in OC.^71,72^ Furthermore, in mouse models of *BRCA*1m BC, two mechanisms of PARPi resistance have been reported: loss-of-53BP1, which is associated with partial restoration of homologous recombination,^73^ and upregulation of genes that encode drug efflux pumps such as MDR1/P-glycoprotein-1.^69,74^

In addition to the monotherapy studies described above, some PARPi are being evaluated in combination with other therapies. While there is promising evidence of improved response in *BRCA*1/2m carriers,^60,75–80^ this approach is challenging due to the associated dose-limiting myelosuppression.^78,79,81^ The BROCADE 2 (NCT01506609),^82^ and BROCADE 3 (NCT02163694)^83^ studies, which are randomised phase II and phase III investigations, respectively, are comparing paclitaxel and carboplatin with or without the PARPi veliparib in *BRCA*1/2m carriers with metastatic BC. These two studies may help characterise the role of combination therapy with PARPi. In addition, a new area of intense active research is the combination of PARPi with immune therapies to treat *BRCA*1/2m-related BC. The phase II MEDIOLA study reported that 20/25 patients with g*BRCA*1/2m and metastatic BC had disease control at 12 weeks under the combination of olaparib with the immunotherapy drug durvalumab.^84^ Furthermore, the National Cancer Institute is currently recruiting patients for a randomised phase II study (NCT02849496) evaluating veliparib and atezolizumab, either as single agents or in combination, for treating patients with stage III–IV *BRCA*-related TNBC. Additional PARPi/immunotherapy studies are in development.

### BRCA mutations in early-stage BC management

Although, *BRCA* status has not strongly influenced outcomes for stage I–III BC, the ability to identify *BRCA*-related cancers and the development of targeted therapies may lead to improved responses. Several studies have evaluated platinum salts in early-stage *BRCA*-related BC, mainly in patients with TNBC, and there are ongoing studies evaluating PARPi in this setting.

### Clinical outcomes to platinum salts in early-stage BC

Byrski et al.^85^ reported pathologic complete response (pCR) to neoadjuvant cisplatin in 65 of 107 women (61%) who had stage I–III TNBC and *BRCA*1m (Table 1). Although their study helped demonstrate the activity of cisplatin in *BRCA*1-related early BC, the patient population had extremely favourable characteristics, with 65% of women having node-negative disease and 46% having tumours ≤2 cm.^85^ Furthermore, it was a single-arm study and did not compare neoadjuvant cisplatin to standard anthracycline-based chemotherapy; that question is being addressed by the ongoing randomised neoadjuvant INFORM/TBCRC 031 study (NCT01670500).

The GeparSixto/GBG 66 study (Table 1) evaluated the addition of carboplatin to standard paclitaxel, doxorubicin, and bevacizumab as neoadjuvant chemotherapy in previously untreated patients with stage II–III TNBC (as well as HER2-positive BC).^86,87^ Patients with TNBC who received additional carboplatin had significantly higher pCR rates and longer disease-free survival;^88^ however, a preplanned sub-analysis by g*BRCA*1/2m status noted the unexpected finding that the benefits of adding carboplatin were only significant in patients with wild-type *BRCA*1/2 (pCR 55.0% with carboplatin versus 36.4% without carboplatin; *P* = 0.004).^88^ No significant pCR benefit was observed in the 50 patients with g*BRCA*1/2m (65.4% with carboplatin versus 66.7% without carboplatin; *P* = 0.92). Analysis of disease-free survival was similar, with carboplatin only improving disease-free survival in patients with wild-type *BRCA*1/2. One potential explanation for these results is that anthracycline-based chemotherapy was already effective in patients with *BRCA*-related BC, owing to the inability of the tumour cells to repair anthracycline-induced DNA damage.^88^ Alternatively, the number of mutation carriers in the study may have been too small to permit detection of a treatment difference. The CALGB 40603 study also reported improved pCR with the addition of carboplatin to neoadjuvant therapy in stage II–III TNBC, although an analysis of response according to *BRCA*1/2m status has not been reported.^81,89^ Thus, the role of *BRCA* testing to guide chemotherapy selection in this setting remains unclear.

### Clinical outcomes to PARP inhibitors in early-stage BC

A feasibility study of neoadjuvant talazoparib administered prior to neoadjuvant chemotherapy in patients with g*BRCA*1/2m and HER2-negative BC reported decreases in tumour volume (median 88%; range 30–98%) in all 13 patients after 2 months of therapy.^90^ These promising results led to the ongoing phase II study of single-agent talazoparib, with results expected in 2018 (NCT02282345).^91^ In addition, several PARPi are currently in phase II/III studies for adjuvant or neoadjuvant therapy in women with TNBC or HER2-negative BC and g*BRCA*1/2m, as described in Table 3.^91–93^

**Table 3:** Selected ongoing phase II/III studies with PARP inhibitors in g*BRCA*1/2m early-stage breast cancer

Study	Disease	Phase	*N*	Treatment	Status
OlympiA NCT02032823^92^	g*BRCA*1/2m with either TNBC or ER+ and/or PgR+ and HER2-negative BC	III	1500 (est)	Olaparib tablets vs placebo after surgery and at least 6 cycles of neoadjuvant or adjuvant chemotherapy	ONGOING
NCT02282345^91^	g*BRCA*1/2m stage I–III BC ≥1 cm, HER2-negative	II	36	Neoadjuvant talazoparib 1 mg (monotherapy) up to 6 months	ONGOING
BRE09-146 NCT01074970^93^	TNBC or g*BRCA*1/2m HER2-negative BC with residual disease after neoadjuvant chemotherapy	II	135	Cisplatin IV every 21 days for 4 cycles vs cisplatin (same dose) plus rucaparib on days 1, 2, 3 every 21 days for 4 cycles	ONGOING

*BC* breast cancer, *ER+* oestrogen receptor-positive, *est* estimated, *gBRCA1/2m* germline *BRCA* mutation, *HER2* human epidermal growth factor receptor 2, *PgR+* progesterone receptor-positive, *TNBC* triple-negative BC

### Local management and the role of prophylactic surgery

The risk of local recurrence after excision and radiotherapy is similar in patients with *BRCA*1/2m BC and those with sporadic BC.^2,6,94–96^ Due to the ability of ionising radiation to induce DS breaks in DNA, and the involvement of *BRCA*1 and *BRCA*2 in repair of DS DNA, it has been suggested that *BRCA*1/2m-related tumours may be susceptible to the therapeutic and toxic effects of radiotherapy. However, no increased local reaction to radiation therapy has been observed in *BRCA*1 and *BRCA*2 mutation carriers with BC. Current guidelines indicate that patients with *BRCA*1/2m-related BC are not eligible for accelerated partial breast irradiation,^19^ but that is based on lack of data rather than evidence for increased toxicity or decreased efficacy with accelerated partial breast irradiation; patients with *BRCA*1/2m-related BC were excluded from accelerated partial breast irradiation studies because they carry a high risk for second primary BC.

In healthy women with *BRCA*1/2m, preventive salpingo-oophorectomy is recommended to reduce the risk of OC during the fifth decade of life.^19,97,98^ The effects of salpingo-oophorectomy on preventing BC in healthy premenopausal women with *BRCA*1m remains controversial,^99,100^ although the benefits in *BRCA*1/2m carriers are established. In premenopausal women with a recent diagnosis or history of BC and *BRCA*1/2m, several large studies have demonstrated that oophorectomy confers strong reductions in risk of subsequent BC, as well as mortality.^100–106^ Metcalfe et al.^105^ studied the benefits of oophorectomy soon after a diagnosis of BC in *BRCA*1/2m carriers (mean age at diagnosis ~42 years). In women with a new primary diagnosis of BC who had bilateral oophorectomy, the adjusted HR for BC-related death was 0.38 (95% CI: 0.19–0.77) for *BRCA*1m carriers and 0.57 (95% CI: 0.23–1.43) for *BRCA*2m carriers. Interestingly, the reduction in risk associated with oophorectomy was greater in women with ER-negative BC (HR 0.07; 95% CI: 0.01–0.51) than in women with ER-positive BC (HR 0.76; 95% CI: 0.32–1.78).^105^ On the basis of these findings, the authors recommend that premenopausal women with newly diagnosed BC be tested for *BRCA*1/2m and, if mutation-positive, offered salpingo-oophorectomy as part of their treatment plan; however, this approach is controversial and not widely adopted.

## Hormone receptor status, *BRCA* mutation status, and response to PARP inhibitors

A multinational epidemiologic study characterised the receptor status of breast tumours from women with g*BRCA*1m (*n* = 3797) or g*BRCA*2m (*n* = 2392)^107^. Among *BRCA*1m carriers, 22% of tumours were ER-positive and 21% were PgR-positive, and among *BRCA*2m carriers, 77% were ER-positive and 64% were PgR-positive. There is evidence, however, that ER-positive tumours arising in *BRCA*1/2m carriers have different morphologic features than ER-positive tumours arising in non-carriers, with those in carriers more frequently exhibiting a higher histologic grade, higher oncotype recurrence score, and more often classified as luminal B by gene expression profiling.^16,107–109^ Furthermore, the percentage of ER-positive BC increases with increasing patient age in *BRCA*1m carriers, but decreases with increasing age in *BRCA*2m carriers.^16,107^

Although a substantial percentage of patients with *BRCA*1/2m BC are also hormone receptor-positive, the influence of hormone receptor-positive disease on DNA-targeted therapy efficacy is still being evaluated. One clue may come from observations concerning loss-of-heterozygosity of the wild-type allele in *BRCA*1 carriers, which is observed at a similar rate in ER-positive and ER-negative tumours, thereby suggesting a similar degree of impairment to the homologous recombination pathway.^15,16^ Clinical data to address this question are limited, but three olaparib studies reported outcomes in patients with ER-positive BC. In the ICEBERG 1 study (NCT00494234), 12 patients in the 400 mg olaparib arm had ER-positive disease and treatment responses were observed.^57^ In another study (Study 42), responses to olaparib as late-line therapy were observed in 4 of 32 patients (12.5%) with ER-positive disease and 4 of 30 (13.3%) with ER-negative disease.^58^ The phase III OlypmiAD study enroled patients with either hormone receptor-positive or TNBC. Recently, exploratory sub-group analyses for PFS demonstrated that the hazard ratio favoured olaparib versus chemotherapy for *BRCA* carriers of either subtype.^61^ In addition, the ABRAZO study showed similar response rates to talazoparib for *BRCA*1/2m carriers with metastatic ER-positive and TNBC: 29% and 26%, respectively.^65^ Although larger studies are needed, these results indicate that ER-positive BC in *BRCA*1/2m carriers are responsive to PARPi.

## Beyond *BRCA* mutations: *BRCA*ness and scoring systems

Another evolving aspect of testing to predict responsiveness to agents that interfere with DNA repair is the use of assays or scoring schemes that indicate the activity level of the homologous recombination pathway.^110^ Tumours that exhibit clinical and biological features of g*BRCA*1/2m disease, despite not having a g*BRCA*1/2m, are thought of as having *BRCA*ness.^111^ BRCA1/2 proteins are essential components of the homologous recombination pathway for repair of DS breaks in DNA; loss-of-homologous recombination in the absence of a g*BRCA*1/2m represents one form of *BRCA*ness. For example, *BRCA*ness may arise as a result of somatic loss or epigenetic silencing of tumour *BRCA* in the absence of g*BRCA*1/2m.^43^ In addition, numerous other proteins participate in the homologous recombination pathway, and mutations in their corresponding genes may also confer *BRCA*ness and subsequently, response to platinum agents or PARPi.

Current data support the hypothesis that tumours deficient in homologous recombination are susceptible to PARP inhibition.^39^ For example, olaparib demonstrated activity in a phase II study in patients with metastatic, castration-resistant prostate cancer and germline or somatic mutations in DNA repair genes.^27^ Among 49 evaluable patients, 16 (33%) had a response and 12 remained on therapy for >6 months. Post hoc analysis in prospectively collected biopsy samples demonstrated a response to single-agent olaparib in 14 of 16 patients who were biomarker-positive, defined as having a homozygous deletion or biallelic deleterious mutation of a DNA repair gene (e.g., *BRCA*2*, ATM, CHEK2*). Among biomarker-negative patients, only 2 of 33 (6%) had a response to olaparib.^27^

Rather than sorting the tangle of how each genetic variation might influence responsiveness to treatment, research has focused on the development of scoring systems intended to quantify the competency of the homologous recombination pathway. Currently there are three prominent scoring systems: homologous recombination defect large-scale transition (HRD-LST), HRD-loss-of-heterozygosity (HRD-LOH), and HRD-telomeric allelic imbalance (HRD-TAI).^112–114^ In the TBCRC 009 study, the sum of the HRD-LOH and HRD-LST scores was associated with response to platinum therapy in patients with metastatic TNBC (HRD-LOH/HRD-LST score of 12.68 for responders versus 5.11 for non-responders; *P* = 0.032).^48^ This scoring approach is also being explored in the neoadjuvant setting. The HRD-TAI score^114^ and an unweighted combination of all three HRD scores^115^ predicted responsiveness to neoadjuvant platinum therapies in women with TNBC. By contrast, the HRD score failed to identify which TNBC patients with metastatic disease had a better response to platinum versus docetaxel during the TNT study (discussed earlier),^45^ although analysis was performed on primary rather than metastatic BC samples.

Using a high-depth whole-genome sequencing approach rather than a targeted sequencing approach, another tool called HRDetect was developed as a predictor of *BRCA*1 and *BRCA*2 deficiency based on mutational signatures. A lasso logistic regression model identified six distinguishing mutational signatures and HRDetect identified *BRCA*1/2-deficient tumours with 98.7% sensitivity. In addition, in a cohort of 560 individuals with BC, HRDetect identified 22 tumours with somatic loss-of-*BRCA*1 or *BRCA*2 and 47 tumours with functional *BRCA*1/2 deficiency, none of which had mutations detected with standard analysis. Thus, integrating a method such as HRDetect or other deep sequencing approaches into the clinical assessment process could potentially identify a larger proportion of patients who may have selective therapeutic sensitivity to PARPi.^116^ Recently, an elevated HRDetect score was associated with response to platinum chemotherapy in an observational study of patients with advanced BC;^117^ however, HRDetect has not yet been correlated with therapeutic responses to PARPi. Another recent study evaluated a novel gene expression signature-generating algorithm to predict therapeutic response to PARPi.^110^ The authors demonstrated that the gene expression signatures may be used to identify PARPi-sensitive cancer cell lines, primary patient-derived tumour cells, and patient-derived xenografts. In addition, these gene expression profiles were found to outperform currently accepted clinical biomarkers of response, including *BRCA*1/2m status.

At this time, scoring systems are still investigational and require further validation before they can be considered for routine clinical use. These scoring systems, or similar methods, may hold the promise of a relatively simple way to incorporate the status of *BRCA* mutations, *BRCA* silencing, and non-*BRCA* mutations into a single score that indicates the competency of homologous recombination and predicts response to platinum-based therapies. Further studies are also needed to determine whether any of these systems predict response to PARPi.

## Conclusion

*BRCA*1/2 mutations occur in women with all BC subtypes, but more commonly in those with early onset or a suggestive family history. Recent studies have found evidence that *BRCA*1/2m carriers with BC have high rates of response to platinum salts in the metastatic and neoadjuvant settings; currently, most of the data were derived from *BRCA*1/2m carriers with TNBC. For *BRCA*1/2m carriers with metastatic TNBC, platinum chemotherapy has been shown to be superior to docetaxel in the first-line setting. For *BRCA*1/2m carriers in the early setting, it is not yet clear whether platinum agents are superior to, or provide additional benefit to conventional anthracycline-based or taxane-based chemotherapy. However, for *BRCA*1/2m carriers with newly diagnosed TNBC for whom an anthracycline is contraindicated, a platinum-based regimen is reasonable. For *BRCA*1/2m carriers with hormone receptor-positive BC, standard chemotherapy should likely be used until more data regarding the efficacy of platinum chemotherapy are available.

Evidence also supports *BRCA*1/2m status as a predictor of responsiveness to PARPi in patients with metastatic BC. For *BRCA*1/2m carriers with metastatic disease and fewer than two chemotherapy regimens in the metastatic setting, use of the PARPi olaparib results in a significantly longer PFS and better quality of life than standard chemotherapy. Now that the first PARPi has been approved in BC, olaparib can be recommended for *BRCA*1/2m carriers with HER2-negative metastatic BC. Ongoing studies will address whether PARPi may also provide benefit for *BRCA*1/2m carriers in the early setting, and future studies should seek to determine the relative efficacy of platinum agents compared with PARPi in the metastatic setting, as well as the efficacy of platinum chemotherapy after progression with PARPi.

Studies assessing the benefit of combining PARPi with chemotherapy will need to be compared to treatment with PARPi monotherapy; given the myelosuppression observed when combining PARPi with chemotherapy. These will need to compare continuous treatment with PARPi monotherapy to combinations of chemotherapy with either a reduced dose or intermittent PARPi schedule. Current research is focused on identifying larger subsets of patients with similar responsiveness to these therapies, such as those with de novo somatic *BRCA* mutations, mutations in other genes associated with the homologous recombination pathway for DNA repair, or those exhibiting *BRCA*-associated mutational signatures or other evidence of homologous recombination deficiency.

*BRCA* testing was previously used in BC patients solely to predict the risk of future cancers and guide surgical therapies. As mutation status increasingly informs optimal management of BC, including choice of systemic therapy, efforts to identify all BC patients with germline or somatic *BRCA* mutations will become even more essential. Given the availability of comprehensive and affordable g*BRCA*1/2m testing and the utility of the information for treatment options, it may be reasonable to consider genetic testing more widely than past criteria have allowed. The recent test-to-treat criteria (Box 1) acknowledges the importance of not overlooking patients with metastatic BC who may have g*BRCA*1/2m, and may benefit from PARPi treatment. The recent oncologist-led mainstream approaches to genetic testing may allow greater access to testing and more complete data for these analyses in the near future.^118^

## References

[1] Bordeleau L, Panchal S, Goodwin P (2010). Prognosis of BRCA-associated breast cancer: a summary of evidence. Breast Cancer Res. Treat..

[2] Brekelmans CT, Tilanus-Linthorst MM, Seynaeve C, vd Ouweland A, Menke-Pluymers MB, Bartels CC (2007). Tumour characteristics, survival and prognostic factors of hereditary breast cancer from BRCA2-, BRCA1- and non-BRCA1/2 families as compared to sporadic breast cancer cases. Eur. J. Cancer.

[3] Chen S, Parmigiani G (2007). Meta-analysis of BRCA1 and BRCA2 penetrance. J. Clin. Oncol..

[4] Goodwin PJ, Phillips KA, West DW, Ennis M, Hopper JL, John EM (2012). Breast cancer prognosis in BRCA1 and BRCA2 mutation carriers: an International Prospective Breast Cancer Family Registry population-based cohort study. J. Clin. Oncol..

[5] Kuchenbaecker KB, Hopper JL, Barnes DR, Phillips KA, Mooij TM, Roos-Blom MJ (2017). Risks of breast, ovarian, and contralateral breast cancer for BRCA1 and BRCA2 mutation carriers. JAMA.

[6] Pierce LJ, Levin AM, Rebbeck TR, Ben-David MA, Friedman E, Solin LJ (2006). Ten-year multi-institutional results of breast-conserving surgery and radiotherapy in BRCA1/2-associated stage I/II breast cancer. J. Clin. Oncol..

[7] Rebbeck TR, Mitra N, Wan F, Sinilnikova OM, Healey S, McGuffog L (2015). Association of type and location of BRCA1 and BRCA2 mutations with risk of breast and ovarian cancer. JAMA.

[8] Rennert G, Bisland-Naggan S, Barnett-Griness O, Bar-Joseph N, Zhang S, Rennert HS (2007). Clinical outcomes of breast cancer in carriers of BRCA1 and BRCA2 mutations. N. Engl. J. Med.

[9] Howlader N, Altekruse SF, Li CI, Chen VW, Clarke CA, Ries LA (2014). US incidence of breast cancer subtypes defined by joint hormone receptor and HER2 status. J. Natl Cancer Inst..

[10] Lehmann BD, Bauer JA, Chen X, Sanders ME, Chakravarthy AB, Shyr Y (2011). Identification of human triple-negative breast cancer subtypes and preclinical models for selection of targeted therapies. J. Clin. Invest.

[11] Mayer IA, Abramson VG, Lehmann BD, Pietenpol JA (2014). New strategies for triple-negative breast cancer--deciphering the heterogeneity. Clin. Cancer Res..

[12] Miquel-Cases A, Steuten LM, Retel VP, van Harten WH (2015). Early stage cost-effectiveness analysis of a BRCA1-like test to detect triple negative breast cancers responsive to high dose alkylating chemotherapy. Breast.

[13] Telli M (2015). Evolving treatment strategies for triple-negative breast cancer. J. Natl Compr. Cancer Netw..

[14] Emborgo, T., Muse, K. I., Bednar, E., Oakley, H. D., Litton, J. K., Lu, K. H., et al. Universal BRCA testing and family outreach for women with triple negative breast cancer. *Cancer Res.* **76**,(Suppl 4) (2016) AbstrP2-09-08.

[15] Lips EH, Debipersad R, Scheerman CE, Mulder L, Sonke GS, van der Kolk LE (2017). BRCA1-mutated estrogen receptor positive breast cancer shows BRCAness, suggesting sensitivity to drugs targeting homologous recombination deficiency. Clin. Cancer Res..

[16] Tung N, Wang Y, Collins LC, Kaplan J, Li H, Gelman R (2010). Estrogen receptor positive breast cancers in BRCA1 mutation carriers: clinical risk factors and pathologic features. Breast Cancer Res..

[17] Balmana J, Diez O, Rubio IT, Cardoso F, ESMO Guidelines Working Group. (2011). BRCA in breast cancer: ESMO Clinical Practice Guidelines. Ann. Oncol..

[18] Moyer VA, U. S. Preventive Services Task Force. (2014). Risk assessment, genetic counseling, and genetic testing for BRCA-related cancer in women: U.S. Preventive Services Task Force recommendation statement. Ann. Intern Med..

[19] National Comprehensive Cancer Network. NCCN: Clincial Practice Guidelines in Oncology: Breast Cancer, version 1.2018. Available from: https://www.nccn.org/professionals/physician_gls/f_guidelines.asp.

[20] Couch FJ, Nathanson KL, Offit K (2014). Two decades after BRCA: setting paradigms in personalized cancer care and prevention. Science.

[21] Weigelt B, Comino-Mendez I, de Bruijn I, Tian L, Meisel JL, Garcia-Murillas I (2017). Diverse BRCA1 and BRCA2 reversion mutations in circulating cell-free DNA of therapy-resistant breast or ovarian cancer. Clin. Cancer Res..

[22] Christie EL, Fereday S, Doig K, Pattnaik S, Dawson SJ, Bowtell DDL (2017). Reversion of BRCA1/2 germline mutations detected in circulating tumor DNA from patients with high-grade serous ovarian cancer. J. Clin. Oncol..

[23] Nik-Zainal S, Davies H, Staaf J, Ramakrishna M, Glodzik D, Zou X (2016). Landscape of somatic mutations in 560 breast cancer whole-genome sequences. Nature.

[24] Winter C, Nilsson MP, Olsson E, George AM, Chen Y, Kvist A (2016). Targeted sequencing of BRCA1 and BRCA2 across a large unselected breast cancer cohort suggests that one-third of mutations are somatic. Ann. Oncol..

[25] Hennessy BT, Timms KM, Carey MS, Gutin A, Meyer LA, Flake DD (2010). Somatic mutations in BRCA1 and BRCA2 could expand the number of patients that benefit from poly (ADP ribose) polymerase inhibitors in ovarian cancer. J. Clin. Oncol..

[26] Moschetta M, George A, Kaye SB, Banerjee S (2016). BRCA somatic mutations and epigenetic BRCA modifications in serous ovarian cancer. Ann. Oncol..

[27] Mateo J, Carreira S, Sandhu S, Miranda S, Mossop H, Perez-Lopez R (2015). DNA-repair defects and olaparib in metastatic prostate cancer. N. Engl. J. Med.

[28] Moynahan ME, Chiu JW, Koller BH, Jasin M (1999). BRCA1 controls homology-directed DNA repair. Mol. Cell.

[29] Moynahan ME, Pierce AJ, Jasin M (2001). BRCA2 is required for homology-directed repair of chromosomal breaks. Mol. Cell.

[30] Tutt AN, Lord CJ, McCabe N, Farmer H, Turner N, Martin NM (2005). Exploiting the DNA repair defect in BRCA mutant cells in the design of new therapeutic strategies for cancer. Cold Spring Harb. Symp. Quant. Biol..

[31] Lord CJ, Ashworth A (2012). The DNA damage response and cancer therapy. Nature.

[32] Lee JM, Ledermann JA, Kohn EC (2014). PARP inhibitors for BRCA1/2 mutation-associated and BRCA-like malignancies. Ann. Oncol..

[33] Ashworth A (2008). A synthetic lethal therapeutic approach: poly(ADP) ribose polymerase inhibitors for the treatment of cancers deficient in DNA double-strand break repair. J. Clin. Oncol..

[34] Pommier Y, O’Connor MJ, de Bono J (2016). Laying a trap to kill cancer cells: PARP inhibitors and their mechanisms of action. Sci. Transl. Med..

[35] Murai J, Huang SY, Das BB, Renaud A, Zhang Y, Doroshow JH (2012). Trapping of PARP1 and PARP2 by clinical PARP inhibitors. Cancer Res..

[36] Murai J, Huang SY, Renaud A, Zhang Y, Ji J, Takeda S (2014). Stereospecific PARP trapping by BMN 673 and comparison with olaparib and rucaparib. Mol. Cancer Ther..

[37] Bryant HE, Schultz N, Thomas HD, Parker KM, Flower D, Lopez E (2005). Specific killing of BRCA2-deficient tumours with inhibitors of poly(ADP-ribose) polymerase. Nature.

[38] Farmer H, McCabe N, Lord CJ, Tutt AN, Johnson DA, Richardson TB (2005). Targeting the DNA repair defect in BRCA mutant cells as a therapeutic strategy. Nature.

[39] McCabe N, Turner NC, Lord CJ, Kluzek K, Bialkowska A, Swift S (2006). Deficiency in the repair of DNA damage by homologous recombination and sensitivity to poly(ADP-ribose) polymerase inhibition. Cancer Res..

[40] Bhattacharyya A, Ear US, Koller BH, Weichselbaum RR, Bishop DK (2000). The breast cancer susceptibility gene BRCA1 is required for subnuclear assembly of Rad51 and survival following treatment with the DNA cross-linking agent cisplatin. J. Biol. Chem..

[41] Alsop K, Fereday S, Meldrum C, deFazio A, Emmanuel C, George J (2012). BRCA mutation frequency and patterns of treatment response in BRCA mutation-positive women with ovarian cancer: a report from the Australian Ovarian Cancer Study Group. J. Clin. Oncol..

[42] Gorodnova TV, Sokolenko AP, Ivantsov AO, Iyevleva AG, Suspitsin EN, Aleksakhina SN (2015). High response rates to neoadjuvant platinum-based therapy in ovarian cancer patients carrying germ-line BRCA mutation. Cancer Lett..

[43] Lord CJ, Ashworth A (2016). BRCAness revisited. Nat. Rev. Cancer.

[44] Kilburn LS, TNT Trial Management Group. (2008). ‘Triple negative’ breast cancer: a new area for phase III breast cancer clinical trials. Clin. Oncol..

[45] Tutt, A., Ellis, P., Kilburn, L. S., Gilett, C., Pinder, S., Abraham, J., et al. The TNT trial: a randomized phase III trial of carboplatin (C) compared with docetaxel (D) for patients with metastatic or recurrent locally advanced triple negative or BRCA1/2 breast cancer (CRUK/07/012). Abstract S3-01 presented at the San Antonio Breast Cancer Symposium, San Antonio, TX, 9–13, (2014).

[46] Ellisen, L. W. The BRCA-Like Phenotype in Cancer: Society for Translational Oncology: 2015 Chabner Colloquium: Collaboration in Cancer Trials; 2016 [20 March 2017]. Available from: https://sto-online.org/sites/default/files/2015/STO_Article_CC_Ellisen.pdf.

[47] Byrski T, Dent R, Blecharz P, Foszczynska-Kloda M, Gronwald J, Huzarski T (2012). Results of a phase II open-label, non-randomized trial of cisplatin chemotherapy in patients with BRCA1-positive metastatic breast cancer. Breast Cancer Res..

[48] Isakoff SJ, Mayer EL, He L, Traina TA, Carey LA, Krag KJ (2015). TBCRC009: a multicenter phase ii clinical trial of platinum monotherapy with biomarker assessment in metastatic triple-negative breast cancer. J. Clin. Oncol..

[49] AstraZeneca Pharmaceuticals LP. (2018). LYNPARZA® (olaparib) tablets for oral use [prescribing information revised January 2018].

[50] United States Food and Drug Administration. Letter for accelerated approval of olaparib as monotherapy for patients with deleterious or suspected deleterious germline BRCA mutated (as detected by an FDA-approved test) advanced ovarian cancer who have been treated with three or more prior lines of chemotherapy 2014 [20 June 2017]. Available from: https://www.accessdata.fda.gov/drugsatfda_docs/appletter/2014/206162Orig1s000ltr.pdf.

[51] AstraZeneca Pharmaceuticals LP. (2017). LYNPARZA® (olaparib) tablets, for oral use [prescribing information].

[52] Mirza MR, Monk BJ, Herrstedt J, Oza AM, Mahner S, Redondo A (2016). Niraparib maintenance therapy in platinum-sensitive, recurrent ovarian cancer. N. Engl. J. Med.

[53] Tesaro Inc. (2017). ZEJULA (niraparib) capsules [prescribing information].

[54] United States Food and Drug Administration. Letter for accelerated approval of rucaparib as monotherapy for the treatment of patients with deleterious BRCA mutation (germline and/or somatic) associated advanced ovarian cancer who have been treated with two or more chemotherapies 2016 [20 June 2017]. Available from: https://www.accessdata.fda.gov/drugsatfda_docs/appletter/2016/209115Orig1s000ltr.pdf.

[55] Fong PC, Boss DS, Yap TA, Tutt A, Wu P, Mergui-Roelvink M (2009). Inhibition of poly(ADP-ribose) polymerase in tumors from BRCA mutation carriers. N. Engl. J. Med..

[56] Audeh MW, Carmichael J, Penson RT, Friedlander M, Powell B, Bell-McGuinn KM (2010). Oral poly(ADP-ribose) polymerase inhibitor olaparib in patients with BRCA1 or BRCA2 mutations and recurrent ovarian cancer: a proof-of-concept trial. Lancet.

[57] Tutt A, Robson M, Garber JE, Domchek SM, Audeh MW, Weitzel JN (2010). Oral poly(ADP-ribose) polymerase inhibitor olaparib in patients with BRCA1 or BRCA2 mutations and advanced breast cancer: a proof-of-concept trial. Lancet.

[58] Kaufman B, Shapira-Frommer R, Schmutzler RK, Audeh MW, Friedlander M, Balmana J (2015). Olaparib monotherapy in patients with advanced cancer and a germline BRCA1/2 mutation. J. Clin. Oncol..

[59] Fong PC, Yap TA, Boss DS, Carden CP, Mergui-Roelvink M, Gourley C (2010). Poly(ADP)-ribose polymerase inhibition: frequent durable responses in BRCA carrier ovarian cancer correlating with platinum-free interval. J. Clin. Oncol..

[60] Somlo G, Frankel PH, Arun BK, Ma CX, Garcia AA, Cigler T (2017). Efficacy of the PARP inhibitor veliparib with carboplatin or as a single agent in patients with germline BRCA1- or BRCA2-Associated Metastatic Breast Cancer: California Cancer Consortium Trial NCT01149083. Clin. Cancer Res..

[61] Robson M, Im SA, Senkus E, Xu B, Domchek SM, Masuda N (2017). Olaparib for metastatic breast cancer in patients with a germline BRCA mutation. N. Engl. J. Med.

[62] Delaloge S, Conte PF, Im SA, Senkus-Konefka E, Xu B, Domchek SM (2017). OlympiAD: further efficacy outcomes in patients with HER2-negative metastatic breast cancer and a germline BRCA mutation receiving olaparib monotherapy vs standard single-agent chemotherapy treatment of physician’s choice [abstract 243PD presented at ESMO 2017 Congress]. Ann. Oncol..

[63] Robson M, Ruddy KJ, Senkus-Konefka E, Domchek SM, Masuda N, Delaloge S (2017). OlympiAD: Health-related quality of life (HRQoL) in patients with HER2-negative metastatic breast cancer (mBC) and a germline BRCA mutation (gBRCAm) receiving olaparib monotherapy vs standard single-agent chemotherapy treatment of physician’s choice (TPC) [abstract 290P presented at ESMO 2017]. Ann. Oncol..

[64] Litton, J., Rugo, H. S., Ettl, J., Hurvitz, S., Gonçalves, A., Lee, K.-H., et al. EMBRACA: a phase 3 trial comparing talazoparib, an oral PARP inhibitor, to physician’s choice of therapy in patients with advanced breast cancer and a germline BRCA mutation. San Antonio Breast Cancer Symposium; December 5–9, 2017; abstract GS6-07.

[65] Turner NC, Telli ML, Rugo HS, Mailliez A, Ettl J, Grischke EM (2017). Final results of a phase 2 study of talazoparib (TALA) following platinum or multiple cytotoxic regimens in advanced breast cancer patients (pts) with germline BRCA1/2 mutations (ABRAZO) [abstract presented at 2017 ASCO Annual Meeting]. J. Clin. Oncol..

[66] Tesaro Inc. A. Phase III Trial of Niraparib Versus Physician’s Choice in HER2 Negative, Germline BRCA Mutation-positive Breast Cancer Patients (BRAVO) 2017 [cited 2017 August 22]. Available from: https://clinicaltrials.gov/ct2/show/NCT01905592.

[67] Drew Y, Ledermann J, Hall G, Rea D, Glasspool R, Highley M (2016). Phase 2 multicentre trial investigating intermittent and continuous dosing schedules of the poly(ADP-ribose) polymerase inhibitor rucaparib in germline BRCA mutation carriers with advanced ovarian and breast cancer. Br. J. Cancer.

[68] Patsouris A, Vicier C, Campion L, Gouraud W, Jimenez M, Pezzella V (2017). An open-label, phase II study of rucaparib, a PARP inhibitor, in HER2- metastatic breast cancer patients with high genomic loss of heterozygosity: RUBY [abstract and poster presented at 2017 ASCO Annual Meeting]. J. Clin. Oncol..

[69] Sonnenblick A, de Azambuja E, Azim HA, Piccart M (2015). An update on PARP inhibitors--moving to the adjuvant setting. Nat. Rev. Clin. Oncol..

[70] Stover EH, Konstantinopoulos PA, Matulonis UA, Swisher EM (2016). Biomarkers of response and resistance to DNA repair targeted therapies. Clin. Cancer Res..

[71] Norquist B, Wurz KA, Pennil CC, Garcia R, Gross J, Sakai W (2011). Secondary somatic mutations restoring BRCA1/2 predict chemotherapy resistance in hereditary ovarian carcinomas. J. Clin. Oncol..

[72] Barber LJ, Sandhu S, Chen L, Campbell J, Kozarewa I, Fenwick K (2013). Secondary mutations in BRCA2 associated with clinical resistance to a PARP inhibitor. J. Pathol..

[73] Jaspers JE, Kersbergen A, Boon U, Sol W, van Deemter L, Zander SA (2013). Loss of 53BP1 causes PARP inhibitor resistance in Brca1-mutated mouse mammary tumors. Cancer Discov..

[74] Rottenberg S, Jaspers JE, Kersbergen A, van der Burg E, Nygren AO, Zander SA (2008). High sensitivity of BRCA1-deficient mammary tumors to the PARP inhibitor AZD2281 alone and in combination with platinum drugs. Proc. Natl Acad. Sci. USA.

[75] Balmana J, Tung NM, Isakoff SJ, Grana B, Ryan PD, Saura C (2014). Phase I trial of olaparib in combination with cisplatin for the treatment of patients with advanced breast, ovarian and other solid tumors. Ann. Oncol..

[76] Del Conte G, Sessa C, von Moos R, Vigano L, Digena T, Locatelli A (2014). Phase I study of olaparib in combination with liposomal doxorubicin in patients with advanced solid tumours. Br. J. Cancer.

[77] Oza AM, Cibula D, Benzaquen AO, Poole C, Mathijssen RH, Sonke GS (2015). Olaparib combined with chemotherapy for recurrent platinum-sensitive ovarian cancer: a randomised phase 2 trial. Lancet Oncol..

[78] Rajan A, Carter CA, Kelly RJ, Gutierrez M, Kummar S, Szabo E (2012). A phase I combination study of olaparib with cisplatin and gemcitabine in adults with solid tumors. Clin. Cancer Res..

[79] Samol J, Ranson M, Scott E, Macpherson E, Carmichael J, Thomas A (2012). Safety and tolerability of the poly(ADP-ribose) polymerase (PARP) inhibitor, olaparib (AZD2281) in combination with topotecan for the treatment of patients with advanced solid tumors: a phase I study. Invest New Drugs.

[80] Soliman HH, Anderson M, Han HS (2017). Outcomes in BRCA1/2 breast cancer patients treated with everolimus followed by chemotherapy+/- PARP inhibitors [abstract presented at 2017 ASCO Annual Meeting]. J. Clin. Oncol..

[81] Sikov WM, Berry DA, Perou CM, Singh B, Cirrincione CT, Tolaney SM (2015). Impact of the addition of carboplatin and/or bevacizumab to neoadjuvant once-per-week paclitaxel followed by dose-dense doxorubicin and cyclophosphamide on pathologic complete response rates in stage II to III triple-negative breast cancer: CALGB 40603 (Alliance). J. Clin. Oncol..

[82] Han HS, Dieras V, Robson M, Palacova M, Marcom PK, Jager A (2018). Veliparib with temozolomide or carboplatin/paclitaxel versus placebo with carboplatin/paclitaxel in patients with BRCA1/2 locally recurrent/metastatic breast cancer: randomized phase II study. Ann. Oncol..

[83] AbbVie Inc. A. Phase 3 Randomized, Placebo-Controlled Trial of Carboplatin and Paclitaxel With or Without the PARP Inhibitor Veliparib (ABT-888) in HER2 Negative Metastatic or Locally Advanced Unresectable BRCA-Associated Breast Cancer 2017 [cited 2017 01 September]. Available from: https://clinicaltrials.gov/ct2/show/NCT02163694.

[84] Domchek, S. M., Postel-Vinay, S., Bang, Y.-J., Park, Y. H., Alexandre, J., Delord, J.-P., et al. An open-label, multitumor, phase II basket study of olaparib and durvalumab (MEDIOLA): results in germline BRCA-mutated (gBRCAm) HER2-negative metastatic breast cancer (MBC). San Antonio Breast Cancer Symposium; December 5–9, 2017; abstract PD6-11.

[85] Byrski T, Huzarski T, Dent R, Marczyk E, Jasiowka M, Gronwald J (2014). Pathologic complete response to neoadjuvant cisplatin in BRCA1-positive breast cancer patients. Breast Cancer Res. Treat..

[86] von Minckwitz G, Schneeweiss A, Loibl S, Salat C, Denkert C, Rezai M (2014). Neoadjuvant carboplatin in patients with triple-negative and HER2-positive early breast cancer (GeparSixto; GBG 66): a randomised phase 2 trial. Lancet Oncol..

[87] von Minckwitz G, Loibl S, Schneeweiss A, Salat CT, Rezai M, Zahm DM (2016). Early survival analysis of the randomized phase II trial investigating the addition of carboplatin to neoadjuvant therapy for triple-negative and HER2-positive early breast cancer (GeparSixto) [abstract and slides presented at the 2015 San Antonio Breast Cancer Symposium]. Cancer Res..

[88] Hahnen E, Lederer B, Hauke J, Loibl S, Krober S, Schneeweiss A (2017). Germline mutation status, pathological complete response, and disease-free survival in triple-negative breast cancer: Secondary analysis of the GeparSixto randomized clinical trial. JAMA Oncol..

[89] Helwick, C., Sikov, W., von Minckwitz, G. Role of carboplatin in triple-negative breast cancer still unclear [studies presented at the 2015 San Antonio Breast Cancer Symposium]: The ASCO Post; 2016. Available from: http://www.ascopost.com/issues/january-25-2016/role-of-carboplatin-in-triple-negative-breast-cancer-still-unclear/.

[90] Litton JK, Scoggins M, Ramirez DL, Murthy RK, Whitman GJ, Hess KR (2017). A feasibility study of neoadjuvant talazoparib for operable breast cancer patients with a germline BRCA mutation demonstrates marked activity. NPJ Breast Cancer.

[91] Litton, J. K., Scoggins, M., Whitman, G. J., Barcenas, C. H., Moulder, S. L., Murthy, R. K., et al. A feasibility study of neoadjuvant talazoparib for early-stage breast cancer patients with a germline BRCA pathogenic variant: NCT02282345 [abstract and poster presented at 2017 ASCO Annual Meeting]. *J. Clin. Oncol*. 2017; 35(suppl): abstr TPS595.

[92] Tutt ANJ, Kaufman B, Gelber RD, Mc Fadden E, Goessl CD, Viale G (2015). OlympiA: a randomized phase III trial of olaparib as adjuvant therapy in patients with high-risk HER2-negative breast cancer (BC) and a germline BRCA1/2 mutation (gBRCAm) [abstract presented at 2015 ASCO Annual Meeting]. J. Clin. Oncol..

[93] Hoosier Cancer Research Network. PARP Inhibition After Preoperative Chemotherapy in Patients With Triple Negative Breast Cancer or ER/PR+, HER2 Negative With Known BRCA1/2 Mutations (BRE09-146) 2017. Available from: https://clinicaltrials.gov/ct2/show/NCT01074970.

[94] Robson M, Svahn T, McCormick B, Borgen P, Hudis CA, Norton L (2005). Appropriateness of breast-conserving treatment of breast carcinoma in women with germline mutations in BRCA1 or BRCA2: a clinic-based series. Cancer.

[95] Brekelmans CT, Seynaeve C, Menke-Pluymers M, Bruggenwirth HT, Tilanus-Linthorst MM, Bartels CC (2006). Survival and prognostic factors in BRCA1-associated breast cancer. Ann. Oncol..

[96] Pierce LJ, Phillips KA, Griffith KA, Buys S, Gaffney DK, Moran MS (2010). Local therapy in BRCA1 and BRCA2 mutation carriers with operable breast cancer: comparison of breast conservation and mastectomy. Breast Cancer Res. Treat..

[97] American College of Obstetricians Gynecologists, ACOG Committee on Practice Bulletins--Gynecology, ACOG Committee on Genetics, Society of Gynecologic Oncologists. (2009). ACOG Practice Bulletin No. 103: hereditary breast and ovarian cancer syndrome. Obstet. Gynecol..

[98] National Comprehensive Cancer Network. NCCN: Clincial Practice Guidelines in Oncology: Ovarian Cancer Including Fallopian Tube Cancer and Primary Peritoneal Cancer, version 2.2018. Available from: https://www.nccn.org/professionals/physician_gls/f_guidelines.asp.

[99] Heemskerk-Gerritsen BA, Seynaeve C, van Asperen CJ, Ausems MG, Collee JM, van Doorn HC (2015). Breast cancer risk after salpingo-oophorectomy in healthy BRCA1/2 mutation carriers: revisiting the evidence for risk reduction. J. Natl Cancer Inst..

[100] Kotsopoulos J, Huzarski T, Gronwald J, Singer CF, Moller P, Lynch HT (2017). Bilateral oophorectomy and breast cancer risk in BRCA1 and BRCA2 mutation carriers. J. Natl Cancer Inst..

[101] Metcalfe K, Lynch HT, Ghadirian P, Tung N, Olivotto I, Warner E (2004). Contralateral breast cancer in BRCA1 and BRCA2 mutation carriers. J. Clin. Oncol..

[102] Domchek SM, Friebel TM, Singer CF, Evans DG, Lynch HT, Isaacs C (2010). Association of risk-reducing surgery in BRCA1 or BRCA2 mutation carriers with cancer risk and mortality. JAMA.

[103] Finch AP, Lubinski J, Moller P, Singer CF, Karlan B, Senter L (2014). Impact of oophorectomy on cancer incidence and mortality in women with a BRCA1 or BRCA2 mutation. J. Clin. Oncol..

[104] Huzarski T, Byrski T, Gronwald J, Gorski B, Domagala P, Cybulski C (2013). Ten-year survival in patients with BRCA1-negative and BRCA1-positive breast cancer. J. Clin. Oncol..

[105] Metcalfe K, Lynch HT, Foulkes WD, Tung N, Kim-Sing C, Olopade OI (2015). Effect of oophorectomy on survival after breast cancer in BRCA1 and BRCA2 mutation carriers. JAMA Oncol..

[106] Mavaddat N, Peock S, Frost D, Ellis S, Platte R, Fineberg E (2013). Cancer risks for BRCA1 and BRCA2 mutation carriers: results from prospective analysis of EMBRACE. J. Natl Cancer Inst..

[107] Mavaddat N, Barrowdale D, Andrulis IL, Domchek SM, Eccles D, Nevanlinna H (2012). Pathology of breast and ovarian cancers among BRCA1 and BRCA2 mutation carriers: results from the Consortium of Investigators of Modifiers of BRCA1/2 (CIMBA). Cancer Epidemiol. Biomark. Prev..

[108] Larsen MJ, Kruse TA, Tan Q, Laenkholm AV, Bak M, Lykkesfeldt AE (2013). Classifications within molecular subtypes enables identification of BRCA1/BRCA2 mutation carriers by RNA tumor profiling. PLoS ONE.

[109] Shah PD, Patil S, Dickler MN, Offit K, Hudis CA, Robson ME (2016). Twenty-one-gene recurrence score assay in BRCA-associated versus sporadic breast cancers: Differences based on germline mutation status. Cancer.

[110] McGrail DJ, Lin CCJ, Garnett J, Liu Q, Mo W, Dai H (2017). Improved prediction of PARP inhibitor response and identification of synergizing agents through use of a novel gene expression signature generation algorithm. NPJ Syst. Biol. Appl..

[111] Lim D, Ngeow J (2016). Evaluation of the methods to identify patients who may benefit from PARP inhibitor use. Endocr. Relat. Cancer.

[112] Popova T, Manie E, Rieunier G, Caux-Moncoutier V, Tirapo C, Dubois T (2012). Ploidy and large-scale genomic instability consistently identify basal-like breast carcinomas with BRCA1/2 inactivation. Cancer Res..

[113] Abkevich V, Timms KM, Hennessy BT, Potter J, Carey MS, Meyer LA (2012). Patterns of genomic loss of heterozygosity predict homologous recombination repair defects in epithelial ovarian cancer. Br. J. Cancer.

[114] Birkbak NJ, Wang ZC, Kim JY, Eklund AC, Li Q, Tian R (2012). Telomeric allelic imbalance indicates defective DNA repair and sensitivity to DNA-damaging agents. Cancer Discov..

[115] Telli ML, Timms KM, Reid J, Hennessy B, Mills GB, Jensen KC (2016). Homologous recombination deficiency (HRD) score predicts response to platinum-containing neoadjuvant chemotherapy in patients with triple-negative breast cancer. Clin. Cancer Res..

[116] Davies H, Glodzik D, Morganella S, Yates LR, Staaf J, Zou X (2017). HRDetect is a predictor of BRCA1 and BRCA2 deficiency based on mutational signatures. Nat. Med..

[117] Zhao EY, Shen Y, Pleasance E, Kasaian K, Leelakumari S, Jones M (2017). Homologous recombination deficiency and platinum-based therapy outcomes in advanced breast cancer. Clin. Cancer Res..

[118] George A, Riddell D, Seal S, Talukdar S, Mahamdallie S, Ruark E (2016). Implementing rapid, robust, cost-effective, patient-centred, routine genetic testing in ovarian cancer patients. Sci. Rep..

